# Regional Analysis of Poverty in Ecuador: Sensitivity to the Choice of Equivalence Scales

**DOI:** 10.3389/fpsyg.2022.877427

**Published:** 2022-03-30

**Authors:** Diego F. García-Vélez, Leidy D. Quezada-Ruiz, María del Cisne Tituaña-Castillo, María de la Cruz del Río-Rama

**Affiliations:** ^1^Department of Economics, Universidad Técnica Particular de Loja (UTPL), Loja, Ecuador; ^2^Universidad Técnica Particular de Loja (UTPL), Loja, Ecuador; ^3^Business Management and Marketing Department, Faculty of Business Sciences and Tourism, University of Vigo, Ourense, Spain

**Keywords:** poverty measurement, equivalence scales, poverty line, monetary poverty, Ecuador

## Abstract

Economies of scale and equivalent consumption units, which are present in households, must be considered in the measurement of monetary poverty, in order to obtain indicators that approximate the reality of each household. Therefore, in this research, monetary poverty in Ecuador is measured and analyzed at the provincial level for the period 2009–2016. It works with data from the National Survey of Employment, Unemployment and Underemployment (ENEMDU) and the INEC methodology is used to measure poverty, but per-capita income is replaced by equivalent income, generated by applying a one-parameter scale (Buhmann et al., 1988) and two two-parameter scales (OECD, 1984; Cutler and Katz, 1992). The main results show that monetary poverty rates are significantly lower when equivalent income is applied, that there is high poverty sensitivity depending on the equivalence scale used and that the provinces with the highest levels of poverty are located in the Amazon region.

## Introduction

There is a great diversity of conceptions regarding the definition of poverty (Laverde Rojas et al., 2019), which have evolved over time, changing the notion of who is considered poor (Spicker et al., 2009). In this regard, Laverde Rojas et al. (2019, p. 71) state that “there are currently consensus scenarios where poverty is understood as a result of socioeconomic relations within cultural, legal and political spheres in which people are deprived or excluded from assets and opportunities to which all human beings are entitled to.”

Townsend (1962) argues that poverty is a relative deprivation of goods imposed by a changing society. Thus, the individual or the family is in a situation of poverty when its resources are below the standards of the society in which it is immersed. Later, he defines it as “the situation lived by those whose resources do not allow them to meet social demands and customs assigned to citizens in a given time-space coordinate” (Townsend, 2003, p. 446). Sen (1992, p. 311) defines poverty as “the absence of basic capabilities that allow any individual to integrate into society, through the exercise of his or her will.” These definitions lead to different one-dimensional poverty measurement methods, in which income or consumption is used to construct different indicators, and multidimensional ones, which groups the approach of needs, capabilities and human rights together (Laverde Rojas et al., 2019, p. 73).

Several authors such as Sen (1976), Foster et al. (1984), Hagenaars (1986), and Ravallion (1992) converge on the definition of monetary poverty. They argue that it is necessary to define a minimum welfare standard in order to survive in the surrounding culture. Therefore, the first requirement to obtain monetary or economic poverty is to specify the concept of the poverty threshold or line and the other requirement is to delimit the population’s per-capita income, since a person is considered poor if his or her per-capita income is below the poverty line. This methodology is the one used by the National Institute of Statistics and Censuses (INEC) to identify the population living in monetary poverty in Ecuador.

One of the most relevant definitions of poverty is the one proposed by the World Bank (1990, p. 324), which defines it from a multidimensional approach as “the impossibility of achieving a minimum standard of living in terms of health services, drinking water and education.” Thus, poverty becomes a major concern in developing countries, both due to its impact on individuals’ wellbeing and its effects on economic growth. According to the World Bank (2018a,b), in 1990, the extreme poverty rate, defined as the situation of those who live on less than $ 1.90 a day (PPP- Purchasing Power Parity of 2011) (%), stood at 36% and by 2015 that percentage had decreased to 10%. These data show a great progress in the fight against poverty. However, this rate means that there were 736 million people living in precarious conditions in the world in that year.

According to the World Bank’s (2018c) Poverty and Shared Prosperity Report, which is published every 2 years, it is expected to affect between 9.1 and 9.4% of the world’s population in 2020. This would represent a regression to the 9.2% rate in 2017. If the pandemic had not disrupted the world, the poverty rate would have fallen to 7.9% in 2020, as predicted (World Bank, 2020a). However, the latest World Bank (2020b) report indicates that the pandemic and the economic crisis could put an end to nearly two decades of continuous progress in fighting global poverty. It is estimated that by 2021 the number of people living in extreme poverty could have increased by 150 million people, increasing the extreme poverty rate to 9.4%. The profile of the new poor would even be different from before that of the pandemic.

Specifically, in Latin America and the Caribbean, ECLAC (Economic Commission for Latin America and the Caribbean) mentions that for the year 2018, the poverty rate in Latin America was 30.1% and the extreme poverty rate was 10.7%, indicating that approximately 185 million people lived in poverty and 66 million people in extreme poverty, and this under monetary poverty measures (ECLAC, 2019). However, if these data are compared with those shown for 2014, there are increases of 2.3 and 2.9 percentage points (pp) for poverty and extreme poverty, respectively. The INEC (2022) of Ecuador indicates that for the year 2019, the percentage of people living below the poverty line was 25%. However, this rate increased to 33% in 2020 due to the impact of the pandemic. This demonstrated the decline of a decade in terms of the improvement achieved in Ecuador in the fight against poverty, which had already been forecasted in studies such as those by Correa-Quezada et al. (2020). However, by the end of 2021, the monetary poverty rate decreased to 27.7%, largely due to the country’s economic recovery. This leaves the certainty that poverty is a problem that still requires greater efforts to be fought at global, regional and national levels.

In this framework, the aim of this research is to measure monetary poverty in Ecuador at the provincial level, but unlike the official measure, it seeks to obtain empirical evidence about the sensitivity of poverty measurements when considering economies of scale and equivalent consumption units in households. It works with data from the National Survey of Employment, Unemployment and Underemployment (ENEMDU) for the period 2009–2016 and the INEC methodology is used for measuring poverty, but per-capita income is replaced by equivalent income, generated by applying a one-parameter scale (Buhmann et al., 1988) and two two-parameter scales (OECD, 1984; Cutler and Katz, 1992).

In contrast to studies such as those by Alonzo and Mancero (2011), Chávez and Medina (2012) and Cuesta (2014), who apply equivalence scales in the measurement of poverty at the national level, this paper shows evidence at the provincial level and the sensitivity of the results in the evolution of monetary poverty over 7 years can be observed. These results contribute to the understanding of the phenomenon at the regional level, by incorporating the differences in the composition of households into the analysis, which is useful information for designing social programs to combat poverty.

This document is structured in five sections. After this brief introduction, which contextualizes the subject under study and sets out the objective, the second section presents a review of the literature on monetary poverty, the equivalence scales, and the empirical evidence in this regard. The third section presents the methodology applied and details the data. Section “Analysis of the Evolution of Poverty in Ecuador at the Provincial Level, Using Per-capita Income” analyses and discusses the results on the evolution of poverty.

## Literature Review

Quantitative scientific studies on poverty originated in the studies carried out by Charles Booth and Seebohm Rowntree at the end of the nineteenth century. Later their study advanced toward the design of one-dimensional indexes such as those presented by Watts (1968), 1976, Kakwani (1980), 1983, and Foster et al. (1984), among others. In general, the vision of poverty has been conceived as the impossibility of satisfying certain basic needs, for which the different indexes measure poverty mainly in terms of income, expenses or possession of goods. At present, the study of poverty has advanced toward the multidimensional poverty approach, with contributions from new measurements such as those proposed by Alkire et al. (2010), Alkire and Foster (2011), 2015, and Ciani et al. (2019). However, the monetary approach is still one of the most widely used in identifying the poor, since income is an instrument that improves quality of life.

### Monetary Approach

According to Sen (1976), in order to calculate poverty using the income method, the first requirement is to delimit the poverty line, which comprises all the basic needs of the people that make up a household. It is considered relevant to calculate the poverty line because it indicates that the minimum needs for the people who make up a household are satisfied and if these needs are not satisfied, the household is in a situation of poverty. For Hagenaars (1986), poverty is a situation in which the wellbeing of a household is generated by the willingness to obtain goods and resources, which if are unable to be obtained, falls below the poverty line, which represents the minimum wellbeing of society.

Foster et al. (1984) identify poverty as economic, since this problem depends on the allocation of people’s resources. They also mention that variations in the size and structure of the household influence their poverty levels. Therefore, a standard of the poverty threshold comprises the resources necessary to have a minimum wellbeing and that family who does not have these resources is considered poor. Ravallion (1992) mentions that poverty exists in a society when one or more people do not achieve the level of economic wellbeing to be included in the minimum standard of society; since the wellbeing of households, according to income, is required to be based on the number of members that make up the household. Therefore, two households with the same income level will have a different level of wellbeing if n people live in one and only one person in the other, i.e., the number of people that make up a household influences the possibility of falling into poverty.

In summary, these authors converge on the definition of monetary poverty; in that it is necessary to define a minimum standard of wellbeing or necessary resources in a society in order to survive in the culture that protects and surrounds them. Therefore, the first requirement for calculating economic or monetary poverty is to specify the concept of the poverty threshold or line.

### Poverty Line

According to Ravallion (1998), the poverty line “is the monetary cost of a reference level of wellbeing for a given person, at a given time and place” (p. 117). This author specifies that if people reach society’s minimum wellbeing, they are not considered poor and, on the contrary, if people are below the poverty line they are in a situation of poverty, i.e., the poverty line represents the minimum income level considered necessary to be able to achieve an adequate standard of living in the society in which one lives. This line is constructed by identifying the total cost of all the basic resources that an average adult consumes in a year. This methodology is based on the assessment of the minimum needs and expenses required to maintain an acceptable life.

There are also other ways of identifying the poverty line, such as the one discussed by Atkinson (1975), which consists of establishing it as a fraction of the mean income or the one discussed by Szal (1977), which considers income distribution. Altimir (1979) argues that the most sophisticated method for drawing poverty lines is based on Engel curves, since they relate expenditure on food, clothing and housing with total income. This method involves postulating the proportion of income spent on basic consumption items, according to actual spending patterns, as an indicator of the relative wellbeing of households, and selecting accordingly which proportion of spending on basic items represents minimally acceptable situations.

The method with the longest tradition for quantifying minimum standards of basic needs in terms of income or consumption expenditure is the one proposed by Rowntree (1901). This method consists of normatively establishing in detail the minimum quantities of supplies to satisfy each need or group of needs considered basic, and converting them into quantities of the specific goods required and valuing them at the prices faced by households. The starting point consists of a detailed determination of the cost of a normatively set minimum food requirement for each household composition. Thus, the poverty line can be estimated by the ratio of expenditure on food, clothing and housing to total income.

With regard to this poverty line, there are also other approaches such as the one put forward by Ravallion (2015), which refers to the subjective and objective poverty line:

• Subjective poverty line: it uses the value judgment criterion that each individual or family has to assess its own situation. Those individuals or families who do not meet their expectations of basic needs are defined as poor.
• Objective poverty line: it assesses poverty by using external criteria that are unique to all individuals or families. It specifies that those individuals or families who do not reach the minimum wellbeing of society according to the *a priori* criterion (income or consumption) used to delimit the poverty line are considered poor.

On the other hand, the World Bank (2020b) uses a poverty line that measures the cost of basic needs of food, clothing and housing, for which it applies purchasing power parity to obtain comparable poverty lines for all countries. This line as of 2015 is USD 1.90 per day, although it also considers two higher poverty lines: USD 3.20 and USD 5.50 per day, which are generally applied to measure poverty in middle-income countries (low and high). Similarly, INEC (2022) uses a poverty line that is based on the minimum disposable income level that a person requires to reach a minimum standard of living, which is updated annually using the consumer price index and was USD 85.60 for 2021. In addition, there is also an extreme poverty line that includes only the food component and was USD 48.24 for the same year.

As shown, there are several definitions and approaches to measure poverty. However, for the present study we work with monetary or income poverty. This is understood as the insufficient income of an individual or family to satisfy basic needs. The objective poverty line is used to avoid subjectivity in the measurement, so those people whose per-capita income is lower than this line are considered to be poor.

### Equivalence Scales in Poverty Measurement

The equivalence scales are an adjustment to household income taking into account the characteristics of its members, thus overcoming the weakness of using per-capita income as a measure of wellbeing, since, according to Mancero (2001) and Domínguez Domínguez and Martín Caraballo (2006), per capita income has two drawbacks: it assumes that all the people in the household have exactly the same needs and it overestimates the economies of scale of numerous larger families. Therefore, the equivalence scales adjust the measurement of poverty so that households can be compared, assuming that the ability to possess a level of wellbeing within each household will be conditioned by the characteristics of its members. Therefore, in this research, poverty will be measured by replacing per-capita income with the equivalent income obtained by applying equivalence scales.

Equivalence scales make it possible to compare households with different structures. Therefore, some countries such as the United States, Uruguay and Chile suggest their use and it is also a standard recommended by international organizations such as the Organization for Cooperation and Development (OECD) and ECLAC. Similarly, the National Institute of Statistics of Spain (INE, 2006) maintains that equivalence scales are based on economies of scale and equivalent consumption units. In addition, economies of scale in a household indicate that, with an increase in the number of people in the household, goods and spaces can be shared due to the arrival of a new member of the family and the same wellbeing can be maintained.

In this regard and, on the contrary, Duclos and Mercader-Prats (1999) state that equivalence scales convert family income into income comparable with other individuals, representing the characteristics of the people in the household, one of them being size. Therefore, a large household has greater needs than a smaller one, i.e., the number of people does influence family wellbeing.

However, the Rio Group (2007) states that “unfortunately no one knows precisely how needs vary with respect to family size and composition” (p. 33). This is the reason for the importance of equivalence scales, which aim to approximate a reality, expressing the sensitivity of poverty to the composition and structure of the household.

ECLAC (2000) indicates that one way of classifying equivalence scales for measuring poverty is as follows:

• Behavioral scales: they are estimated from the observed household expenditure.
• Parametric scales: they are calculated based on a functional form, with explicit parameters that reflect the degree of economies of scale and equivalence per consumer unit, of household members.
• Expert scales: they are built based on the criteria of researchers (or experts).
• Subjective scales: they are estimated based on people’s subjective perception of their needs and necessary expenses according to demographic composition.

Behavioral and subjective scales are similar since they are both estimated empirically from household surveys. On the other hand, parametric and expert scales may correspond to the same source of information, which would be experts’ criterion, although parametric scales could be developed by taking into account other sources.

On the other hand, when applying equivalence scales to measure income poverty, each person is not considered with the same weight when distributing income (Pérez, 2008). In fact, this type of adjustment is made based on the number of people in each family, thus obtaining the equivalent income. There is a wide variety of methodologies for applying parametric equivalence scales, which are explained below by grouping them into one-parameter and two-parameter scales.

One-parameter equivalence scales, as the name suggests, are those that depend on a single parameter, especially household size and its elasticity to the equivalence parameter. These scales include the sensitivity of the equivalence parameter to economies of scale, since households can share goods and services when increasing their size and, in turn, maintain family wellbeing. In this type, we identify the scales proposed by Engel (1895), Buhmann et al. (1988), among others.

Authors who follow two-parameter equivalence scales point out that a limitation of one-parameter equivalence scales is that they depend only on household size and not on household composition and other relevant household characteristics. Thus, Coulter et al. (1992) suggest that it is not appropriate to treat the household income, for example, of three adults, the same as that of households that are made up of a single parent, with one or two children, so they assume that an extension should be made, dividing the household components according to whether they are adults or children. The scales proposed by the OECD (1982, 1984), Cutler and Katz (1992), and Deaton and Zaidi (2002), among others, stand out in this type.

### Equivalence Scales in the International and National Context

At the international level, there is a relevant number of applications of equivalence scales, which consider both two-parameter and one-parameter scales, mainly showing the importance of considering the economies of scale that exist in households and the differences in consumption between children and adults. Table 1 summarizes the methodology used and the main results for each investigation considered in the international empirical evidence.

**TABLE 1:** Applications of equivalence scales at the international level.

Authors	Objective and methodology	Results
Kakwani, 1977	The objective is to estimate consumption by household composition. For this study, the Barten scale was applied and data from the Australian Survey of Consumer Finance and Expenditure 1967–1968 were used.	When considering consumption according to group composition, families whose income is higher than that of the standard family are family group 1 (head of household) and 2 (homemaker); on the other hand, homemakers with one child or more are in a situation of poverty, since their consumption is lower than that of the average family.
Buhmann et al., 1988	For ten countries (Australia, Canada, Israel, the Netherlands, Norway, Sweden, Switzerland, the United Kingdom, the United States and West Germany), the study used the equivalence scale of the same name with data from 1979, 1981, 1982, and 1983.	When using equivalence scales in the measurement of poverty, it was determined that theoretically no scale is considered definitive, but given the sensitivity of poverty elasticity, there is a significant decrease and this was shown to be the case for the countries discussed.
Cutler and Katz, 1992	These authors proposed a two-parameter scale, where it is necessary to differentiate between an adult and a child and it is applied for the year 1988 in the United States.	It is identified that consumption poverty is 3 pp lower than income poverty, which supports that there is a different weighting of consumption between an adult and a child and in turn the incidence of poverty decreases with the use of this equivalence scale.
Citro and Michael, 1995	These authors propose estimating the poverty line for the United States, for which they applied the two-parameter scale in their study in the 1980s.	In this study, it was observed that there is an exaggerated degree of economies of scale between the family made up of two adults and that of one adult. In addition, they inferred that the first child in a household increases the needs to a lesser extent than the second and third one.
Valderrama, 2003	It estimates the economies of scale in households and poverty from data from the 1994–1995 Income and Expenditure Survey for 23 capital cities of Colombia, for which it uses a Working-Leser parametric regression.	As a result, it is confirmed that equivalence scales should be included in the measurement of poverty, because it has been proven that a household made up of 4 members needs less than twice as many goods and services compared to a household made up of 2 members, and that a child’s needs are less than those of an adult according to Engel’s economies of scale of consumption.
García and Prieto, 2007	They discuss the evolution of income distribution in Spain in the period 1985–2002. The study shows the inference of equivalent income according to the scale of OECD (1982, 1984) and Buhmann et al. (1988)	In this study, it was found that equivalence scales do not allow for a stable order of income over time, but they do indicate a significant reduction in poverty.
Domínguez and Núñez, 2007	The situation of Andalusia is explored on the basis of the curves of incidence, intensity and inequality (IID) in the period 1997–2000. In turn, the equivalent family expenses are obtained by applying the scale of Buhmann et al. (1988).	When obtaining the results, it is concluded that the national IID curves are sensitive to the equivalence parameters when applying the Buhmann scale, since they indicate that poverty tends to be higher when the equivalence parameter is lower.
Pérez, 2008	This study identifies poverty according to the equivalence scales for Costa Rica, using data from MECOVI and applying the scales of OECD (1982, 1984) and Cutler and Katz (1992).	The study shows a significant difference when estimating income poverty and comparing it with poverty measured by equivalence scales; it also indicates that per-capita income is insufficient for measuring poverty because it does not include the composition and structure of households.
Carugati, 2009	The equivalence scales are applied for Argentina, using household consumption expenditure data for the period 1996–1997 and applying the Buhmann et al. (1988) scale.	The result was that equivalence scales establish an income level lower than the criterion used by INDEC for a household not to be considered poor.
Bishop et al., 2014	They estimate the subjective equivalence scales for the entire Eurozone, including its individual constituent countries, using European Income and Living Conditions (SILC) data for the period 2004–2007.	Their approach allows them to estimate the marginal cost of a child and as their main results they identify that, for the Eurozone, adding the first child is more costly than adding a third adult and that the marginal cost of children decreases.
Echeverría, 2016	In this study, the Barten (1964), parametric and National Institute of Statistics and Censuses (INEC) scales were applied to obtain poverty measures for Argentina in the period 1990–2015.	The study revealed that adults and children are part of the most sensitive group to the choice of scales, indicating that children’s consumption encourages the construction of equivalence scales.
Jayasinghe et al., 2016	The authors adapt of Pendakur (1999) methodology on the baseline independence test and Engel (1895) income-dependent equivalence scales. In order to determine the extent to which conventional poverty rates require adjustment when economies of scale take food consumption into account.	The research examines the effects of household income on the equivalence scales and whether household preferences satisfy the assumption of independence, concluding that the equivalence scales increase with household income, both at the national level and at the sectoral level (urban, rural and farm), that is, low-income households enjoy greater economies of scale.
Newhouse et al., 2017	Using microdata from 104 surveys collected between 2009 and 2014 in 89 developing countries included in the World Bank’s Global Micro Database (GMD) and using different equivalence scales, they estimate the rate of extreme poverty among children in the developing world.	They succeed in testing the robustness of the differences between child and adult poverty rates, concluding that the child poverty rate is more than twice that of adults on all reasonable two-parameter equivalence scales.
Abanokova et al., 2021	They break poverty down into chronic and transitory components, using equivalence scales constructed from subjective wealth and more than 20 household panel survey data from the Russian Longitudinal Monitoring Survey between 1994 and 2017.	In the study it is found that the elasticity of the equivalence scales are sensitive to the demographic composition of the households, so that the adjustments of the equivalence scales result in lower estimates of the poverty lines. Furthermore, by breaking down poverty into chronic and transitory components, they find that chronic poverty is directly related to the adult scale parameter. But, chronic poverty is less sensitive to the child scale factor compared to the adult scale factor.

*Source: Own elaboration from the cited authors.*

Regarding the application of equivalence scales for welfare measures in Ecuador, works such as those by Alonzo and Mancero (2011) are identified, in which the possibilities of estimating the equivalence scales for 16 Latin American countries, including Ecuador, are shown, using the estimation methods of Engel and Rothbarth, and working with data from household budget surveys. A wide heterogeneity is identified in the results of the application of scales for each country, since they show different degrees of sensitivity to the estimation model and to the data characteristics. Therefore, it is proposed that the application of equivalence scales be done through a parametric scale with a predetermined functional form that only considers economies of scale in consumption.

The research developed by Chávez and Medina (2012), analyses the expenditure of Ecuadorian families, establishing equivalence scales according to household size based on the Engel curve. Data from the Living Conditions Survey (ECV) carried out by the INEC for the period 2005–2006 (INEC, 2006) are used. The main result is that the best way to measure the level of wellbeing in Ecuadorian families is by estimating the costs in the form of an equivalent adult, because by doing so the economies of scale that are present in households are incorporated.

Finally, the work by Cuesta (2014) analyses the evolution of poverty in Ecuador in the period 2007–2013, estimating income per person according to the OECD (1982, 1984) scales and the parametric scale of Buhmann et al. (1988). The main results show a significant decrease in poverty and extreme poverty at a general level, but there are still at-risk population groups, on which social inclusion and anti-poverty policies should be focused.

## Data and Methodology

To calculate poverty rates according to the monetary approach, the databases of the National Survey of Employment, Unemployment and Underemployment (ENEMDU) collected by the INEC for the period 2009–2016 were used. The ENEMDU is representative of the Ecuadorian population at the national and provincial levels, with urban and rural coverage for the entire period, as a reference for the year 2016, the survey was conducted in 30,338 households. In addition, it is the most suitable household survey for the analysis and measurement of monetary poverty in Ecuador, since other instruments such as the Survey of Living Conditions (ECV) or the National Survey of Income and Expenditures (ENIGHUR), do not have periodicity annually. We worked with a provincial disaggregation, which allowed us to obtain results for 23 provinces, except for Galapagos because there is no information available for the study period.

The methodology used in this research to calculate poverty is the one provided by the INEC to calculate monetary poverty, which establishes that those people whose per-capita income is lower than the poverty line value are poor. The methodology used by the INEC is maintained, but the calculation of per-capita income is replaced by the equivalent income obtained through the application of equivalence scales, which results from the ratio between total income and the equivalence parameter. The process to obtain the equivalent income is described below.

Per-capita household income is obtained by using the following equation:


$$X=\frac{Y}{n}$$

Where X is the per-capita household income, Y is the total household income and n is the household size. To apply equivalence scales in the measurement of poverty, it is necessary to replace per-capita income by equivalent income. Therefore, n is replaced by E, which is the equivalence parameter and the equation is as follows:


$$X=\frac{Y}{E}$$

Where X is the equivalent income, Y is the total household income and E is obtained from the equivalence scales shown in Table 2.

**TABLE 2:** Equivalence scales for estimating monetary poverty.

Scale	Acronym*	Equation	Description
Buhmann et al., 1988	IEBRSS	E=N^S^,S ∈ [0,1]	Where: S = 0.74 parameter that summarizes the sensitivity of E. N = household size.
Cutler and Katz, 1992	IECK	E=(A + *pK*)^F^,p,F ∈ [0,1]	Where: A = number of adults. K = number of children aged under 15. p = 0.7 parameter that reflects the cost of a child’s resources. F = 0.7 which is an indicator of the degree of economies of scale.
OECD, 1984	IEOCDEM	E=1 + 0.5(A−1) + 0.3K	Where: A = number of adults. K = number of children aged under 15.

*Equivalent income scale of Buhmann, Rainwater, Schmaus and Smeeding (IEBRSS), equivalent income scale of Cutler and Katz (IECK), and equivalent income modified OECD scale (IEOCDEM).

*Source: Own elaboration based on the authors of the table.*

### Analysis of the Evolution of Monetary Poverty in Ecuador

**FIGURE 1:**
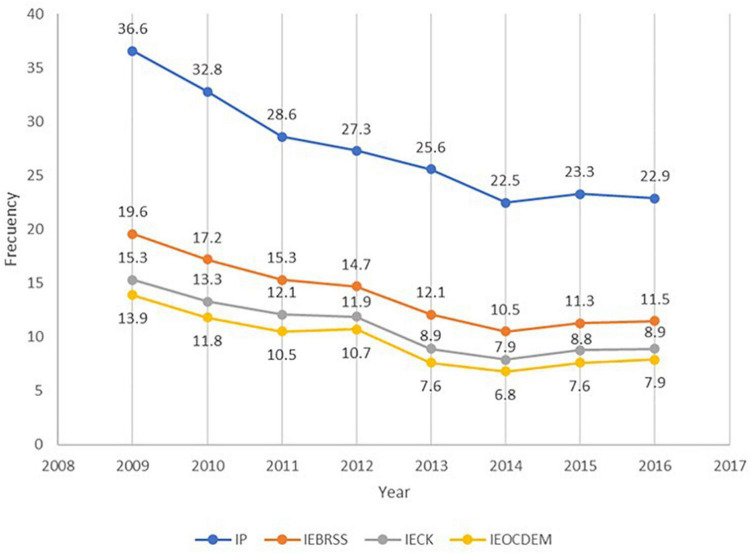
Evolution of the income poverty rate for Ecuador, 2009–2016. Source: Own elaboration with data from the ENEMDU, 2009–2016.

Currently, Ecuador estimates monetary poverty by using per-capita income, considering those people whose per-capita income is below the poverty line as poor. Similarly, when applying income equivalence scales, those whose equivalent income is below the poverty line are considered poor. The results of poverty in Ecuador when using per-capita income (PI) and equivalent income with the application of the OECD (1984), Buhmann et al. (1988), and Cutler and Katz (1992) scales for the period 2009–2016, are shown in Figure 1.

Poverty according to per-capita income and equivalent income obtained by applying equivalence scales shows a decrease from 2009 to 2014. This decrease can be attributed mainly to the country’s economic growth, which averaged 3% during that period, largely due to high oil prices. However, as of 2014, external problems such as the appreciation of the dollar (Ecuador’s official currency) and the fall in oil prices halted the growth of the Ecuadorian economy, which was reflected in income poverty rates, thus demonstrating that structural problems regarding poverty have not yet been solved, and that this indicator fluctuates according to the economic situation. Similarly, Burgos (2013) highlights that an increase or decrease in the oil price largely determines the growth or contraction of the Ecuadorian economy, while he also mentions that economic growth and an increase in the percentage of the employed population are important factors for reducing poverty during this period.

On the other hand, the decrease in poverty for Ecuador in the 2009–2016 period for the PI is 13 pp, for the IEBRSS it is 8 pp and for the IECK and IEOCDEM it is 6 pp. This indicates that the per-capita income measurement shows a greater reduction in monetary poverty in Ecuador throughout this period. In addition, it is evident that there is a high sensitivity of poverty when using equivalence scales, with poverty measured by per-capita income showing the highest rates and poverty measured by the IEOCDEM the lowest rates.

Furthermore, it can be seen that according to each type of measurement, monetary poverty for Ecuador ranges between 13.9 and 36% for 2009 and for 2016, the incidence of income poverty varies between 7.9 and 22.9%, which shows a high sensitivity of poverty depending on the equivalence scale used. In absolute values, if PI is used in Ecuador, then 3.8 million people were in poverty in 2016, and when using IEOCDEM, there were 1.3 million people, which means that 2.5 million people would no longer be considered poor with respect to INEC’s current monetary poverty estimate. When applying equivalence scales, in absolute terms there is a significant decrease in monetary poverty for Ecuador in 2016, since between 1.9 and 2.5 million people would no longer be classified as poor when changing per-capita income to equivalent income.

Similar results are seen in the studies developed by Alonzo and Mancero (2011) and Cuesta (2014), since they identify a wide variety in the results when applying equivalence scales, considering the characteristics of the data and the sensitivity to estimation, in addition to the variation in the percentages of poverty rates.

When analyzing the evolution of monetary poverty at the national level, a general idea of the poverty situation is obtained when applying the PI, IEBRSS, IECK, and IEOCDEM, but a provincial disaggregation is important to determine and compare the sensitivity of the provinces when applying equivalence scales with respect to the official calculation of poverty using PI.

## Analysis of the Evolution of Poverty in Ecuador at the Provincial Level, Using Per-Capita Income

**FIGURE 2:**
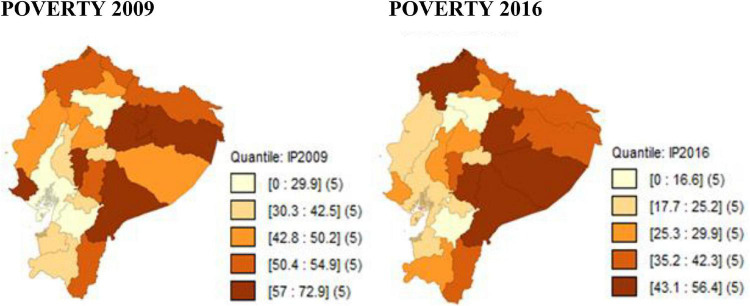
Evolution of poverty in Ecuador at the provincial level, using per- capita income. Source: Own elaboration with data from ENEMDU 2009 and 2016.

Figure 2 shows the evolution of monetary poverty calculated by using per-capita income (PI) in the provinces of Ecuador for the period 2009–2016. In 2009, the provinces with the highest incidence of poverty were: Napo (72.9%), Francisco de Orellana (67.7%), Bolívar (64.9%), and Morona Santiago (62%), which shows that poverty is mainly concentrated in the Amazonian provinces. Similarly, in 2016, the provinces with the highest poverty rate remained the same, but Pastaza (56.4%) and Chimborazo (44%) were added, although poverty continues to be more noticeable mainly in the Amazon region.

Historically, the Amazon has been the region with the highest poverty rate, despite the fact that it has oil reserves and tropical forests. The problems faced by the region are deforestation, extensive cattle raising, monocultures, the disappearance of indigenous peoples and their culture, mining and oil extraction, which do not allow the region to grow. Larrea (2004) confirms that regions with high dependence on oil are characterized by slow economic growth, low productive diversification and a low employment rate, which are conditions that contribute to the persistence of poverty. Similarly, Mideros (2012) and Correa-Quezada et al. (2018) in their research on poverty in Ecuador, identify that the majority of the poor live in the rural areas of the country and proportionally, there are more poor people in the Amazon than in the Costa or Sierra regions.

During the 8 years of the study period, the provinces with the greatest variation in poverty reduction are: Santo Domingo de los Tsáchilas, which decreased from 48.2% (2009) to 16.6% (2016), which means that it decreased by 31.6 pp; In Santa Elena, the incidence of income poverty decreased by 29.4 pp.

On the other hand, the provinces that showed a decrease in income poverty over this period in lower proportion than the other provinces are: Pichincha with a 1.1 pp reduction and Guayas with an 8 pp decrease. This is because the lower the poverty rates, the more difficult it is to reduce poverty, since it is necessary to apply local public policies, according to the reality of each province. A similar characteristic of these provinces is that they are considered poles of economic concentration and poverty for the year 2016 does not exceed 20%.

In general terms, by using PI, monetary poverty for the provinces of Ecuador in 2009 ranged from 29.9 to 72.9%, and for 2016, the incidence of income poverty was between 16.6 and 56.4%, which shows a high reduction in poverty during the study period.

### Analysis of the Evolution of Poverty in Ecuador at the Provincial Level, Using Equivalent Income

As shown by Buhmann et al. (1988) in the application of the IEBRSS equivalence scale for the United States, theoretically no scale is considered definitive, but when faced with the sensitivity of elasticity of poverty, a significant decrease is observed, since there is a large reduction in poverty according to the STAT elasticity (0.7). Similarly, this is true for the provinces of Ecuador in the period 2009–2016, with the application of this scale with 0.7 elasticity.

**FIGURE 3:**
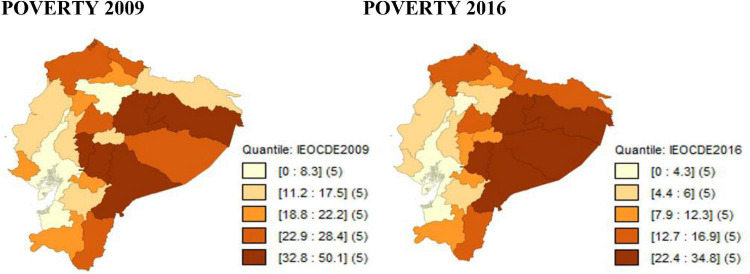
Evolution of poverty by equivalent income with the IEOCDEM scale. Source: Own elaboration with data from ENEMDU 2009 and 2016.

**FIGURE 4:**
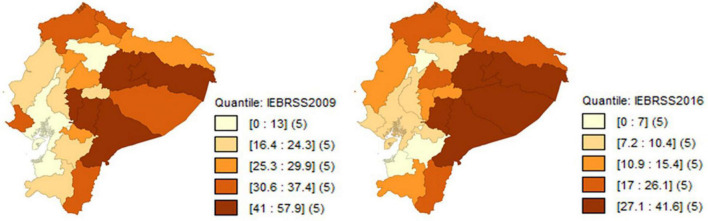
Evolution of poverty by equivalent income with the IECK scale. Source: Own elaboration with data from ENEMDU 2009 and 2016.

**FIGURE 5:**
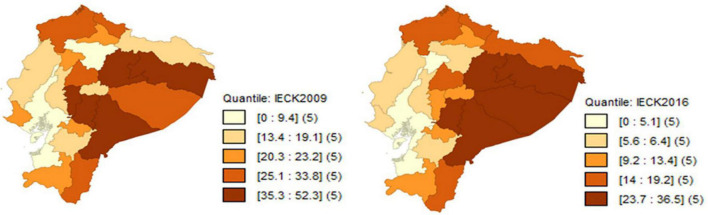
Evolution of poverty by equivalent income with the IEOCDEM scale. Source: Own elaboration with data from ENEMDU 2009 and 2016.

Poverty measured by equivalence scales for the provinces of Ecuador in the 2009–2016 period, decreases significantly in relation to poverty estimated by per-capita income. However, the concentration of poverty continues to be in the Amazon region, as regardless of the equivalence scale used, the provinces with the highest poverty rate are: Francisco de Orellana, Napo, Morona Santiago and Pastaza, which are ironically the provinces that concentrate the country’s oil exploitation, which is the main commodity of the Ecuadorian economy (see Figures 3–5).

When using per-capita income and equivalent income in the poverty measurement, there is a similar characteristic, which is that the provinces with the highest poverty rate in 2009 are: Francisco de Orellana, Bolívar and Morona Santiago, while for 2016 they are: Pastaza, Morona Santiago, Napo and Chimborazo. When using equivalence scales, the decrease in poverty is significant at the provincial level, since for 2009, the poverty rate according to per-capita income for some provinces is higher than 60%, while with the equivalent income, poverty for all provinces is lower than that value, and the same occurs for 2016, but with a limit of 41% for the poverty rate.

In sum, equivalence scales directly influence the measurement of poverty, therefore, poverty according to equivalent income indicates a significant decrease at the provincial level compared to the usual measurement of monetary poverty according to per-capita income.

### Analysis of the Sensitivity of Poverty

In order to have a better perception of the difference between poverty calculated with per-capita income and that calculated with equivalent income, Table 3 shows the variations in percentage points (pp) on the poverty rate at the provincial level.

**TABLE 3:** Sensitivity of poverty in Ecuador at the provincial level, 2009–2016.

Provinces/Scales	Poverty rate 2009					Poverty rate 2016				
		
	IP	IEBRSS	IECK	IEOCDEM	Variation	IP	IEBRSS	IECK	IEOCDEM	Variation
Bolívar	64.9	47.4	41.2	37.9	27.0	36.6	15.2	9.6	7.9	28.7
Esmeraldas	53.9	33.8	26.6	24.9	29.0	43.1	26.1	19.2	16.9	26.2
Santa Elena	57.0	30.6	21.3	19.2	37.8	27.6	8.8	5.6	4.7	22.9
Zamora Chinchipe	51.7	34.0	25.1	22.9	28.8	37.0	21.9	16.9	15.0	22.0
Sucumbíos	50.4	25.3	19.1	17.5	32.8	37.9	23.5	17.6	16.0	21.9
Chimborazo	54.9	41.0	35.3	32.8	22.1	44.0	27.1	23.7	22.4	21.6
Pastaza	50.2	37.4	33.8	28.4	21.8	56.4	41.6	36.5	34.8	21.6
Manabí	42.8	22.2	16.9	14.8	28.0	25.2	10.9	5.6	4.4	20.8
Los Ríos	40.8	19.2	13.4	11.2	29.6	25.3	9.6	6.4	5.6	19.7
Carchi	52.9	33.4	28.8	26.1	26.8	35.2	21.4	17.9	15.7	19.5
Francisco de Orellana	67.7	57.9	52.3	50.1	17.6	42.3	28.7	25.8	23.1	19.2
Morona Santiago	62.0	43.4	40.5	38.0	24.0	50.6	39.1	34.0	32.1	18.5
Napo	72.9	47.6	39.9	39.7	33.2	49.8	39.5	33.9	31.8	18.0
Cotopaxi	48.3	29.9	25.8	24.3	24.0	29.9	17.0	14.0	12.7	17.2
Imbabura	44.4	27.7	23.2	22.2	22.2	27.8	14.8	11.7	10.7	17.1
Cañar	42.5	26.3	20.3	19.1	23.4	24.4	10.4	9.2	8.6	15.8
Loja	42.5	24.3	20.3	18.8	23.7	27.5	15.4	13.4	12.3	15.2
Nacional	36.0	19.6	15.3	13.9	22.1	22.9	11.5	8.9	7.9	15.0
El Oro	30.3	13.0	9.4	8.3	22.0	18.3	6.2	5.0	3.8	14.5
Santo Domingo de los Tsáchilas	48.2	29.2	22.2	18.9	29.3	16.6	7.0	4.1	3.2	13.4
Guayas	25.6	9.6	6.4	5.6	20.0	17.7	7.2	5.1	4.3	13.4
Tungurahua	33.3	18.2	14.2	13.1	20.2	21.8	13.2	11.4	9.9	11.9
Azuay	29.9	16.4	14.7	12.9	17.0	15.0	6.6	6.0	5.3	9.7
Pichincha	14.7	7.0	5.6	5.3	9.4	13.6	7.4	6.3	6.0	7.6

*The data are arranged from the highest to the lowest according to the variation between the poverty rate with PI and IEOCDEM for 2016.*

*Source: Own elaboration with data from ENEMDU 2009 and 2016.*

The results show that the scale with the greatest sensitivity to the equivalence parameter in the measurement of poverty is the IEOCDEM. The reason for this is that the weighting of consumption given to an adult is different from that of a child. It can be seen that the greatest variation in the poverty reduction for 2009 is in the provinces of Santa Elena with 37.8 pp, Napo with 33.2 pp and Sucumbíos with 32.8 pp. While for 2016, the provinces with the greatest variation in poverty reduction are Bolívar, Esmeraldas and Santa Elena, with 28.7, 26.2, and 22.9 pp, respectively.

The results suggest that poverty rates are sensitive to equivalence scales, since there is evidence of changes in the incidence of poverty in favor of certain types of households depending on the scale used. The choice of scale could significantly affect the identification and relative composition of the beneficiaries of social policies.

## Conclusion

The existence of economies of scale and equivalent consumption units in households justify the importance of applying equivalence scales in the measurement of monetary poverty. The former suggests that with an increase in the number of people in the household, goods and spaces can be shared with the arrival of a new family member and the same wellbeing can be maintained. The latter indicates that consumption is different between an adult and a child, since it is considered that an adult tends to have a higher consumption.

In contrast to other research applied in Ecuador (Alonzo and Mancero, 2011; Chávez and Medina, 2012; Cuesta, 2014), the results from applying equivalence scales provide findings at the provincial level in a period of time, which makes it possible to show the differences at the regional level for the most effective application of public policies to combat poverty. In addition, the application of equivalent income is not only useful for developing countries, but also helps in understanding the phenomenon in developed countries, as shown by Domínguez and Núñez (2007); Herrero (2017), and Kuypers and Marx (2018).

By using equivalent income instead of per-capita income, it is evident that the monetary poverty rates are significantly lower, and there is a high sensitivity in poverty according to the equivalence scale used. While it is true that using equivalent income allows for an analysis closer to reality, the lack of evidence to identify the best equivalence scale means that per-capita income continues to be the most widely used in monetary poverty measurements at the global level, such as those developed by ECLAC (2019) and the World Bank (2020b).

Throughout the study period, there has been a clear reduction in poverty at the national and provincial level. However, there is no evidence of changes in terms of reducing the poverty gap between the poorest and the least poor provinces, and the poorest provinces continue to be those located in the Amazon region, which are provinces that have historically been deprived of development despite being the main oil producers in Ecuador.

As for the theoretical implications of this study, it can be seen that although today there is a broad trend toward the study of poverty with a multidimensional approach, the monetary approach continues to be important in national and international poverty measurements. This is because the lack of economic resources could be one of the main reasons for people to be deprived of certain basic needs such as housing, health or education. Furthermore, as practical implications, the use of two-parameter scales in the measurement of monetary poverty is suggested, because they manage to capture economies of scale and differences in consumption between an adult and a child. Therefore, these equivalence scales allow us to get closer to reality and facilitate the distinction between a poor and non-poor household.

Finally, the main limitation of the study lies in the lack of updated information at the provincial level and even at other levels of disaggregation. This would allow conclusions to be drawn at the local level, and which is not possible, since the most current databases available that capture the income of the Ecuadorian population are mainly representative only at the national level.

## Data Availability Statement

The original contributions presented in the study are included in the article/supplementary material, further inquiries can be directed to the corresponding author/s.

## Author Contributions

DG-V, LQ-R, MCT-C, and MCR-R: conceptualization, investigation, methodology, formal analysis, writing—original draft, preparation and writing—review and editing. All authors listed have made a substantial, direct, and intellectual contribution to the work, and read and approved it for publication.

## Conflict of Interest

The authors declare that the research was conducted in the absence of any commercial or financial relationships that could be construed as a potential conflict of interest.

## Publisher’s Note

All claims expressed in this article are solely those of the authors and do not necessarily represent those of their affiliated organizations, or those of the publisher, the editors and the reviewers. Any product that may be evaluated in this article, or claim that may be made by its manufacturer, is not guaranteed or endorsed by the publisher.
